# Type 1 Diabetes Mellitus and Multiple Sclerosis: An Association to Consider

**DOI:** 10.7759/cureus.30762

**Published:** 2022-10-27

**Authors:** Catarina Almeida, Gabriela Venade, Daniela Duarte, Alexandra Vaz, Edite Nascimento

**Affiliations:** 1 Department of Internal Medicine, Centro Hospitalar Tondela-Viseu, Viseu, PRT

**Keywords:** genetic susceptibility, autoimmunity, autoimmune diseases, multiple sclerosis, type 1 diabetes mellitus

## Abstract

Type 1 diabetes mellitus (T1DM) and multiple sclerosis (MS) have been described as chronic organ-specific diseases, approached by different medical specialties. However, they share more etiologic and pathologic features than expected between two autoimmune diseases.

The authors present the case of a 40-year-old Caucasian male, diagnosed with type 1 diabetes mellitus at age 18, with poor metabolic control in the early years after the diagnosis. Fourteen years after the diagnosis of diabetes, he started complaining of paresthesias in both feet and sexual dysfunction. Months later, he began to have episodes of muscle weakness and decreased strength in the right lower limb, with a relapsing-remitting pattern and diplopia. This typical course of the symptoms associated with characteristic findings in brain magnetic resonance imaging, with multiple lesions, with evidence of space and time dissemination, established the diagnosis of multiple sclerosis. The presence of oligoclonal bands in the cerebrospinal fluid analysis sustained this diagnosis. Other alternative etiologies were excluded.

People with type 1 diabetes mellitus are at an increased risk for other autoimmune diseases, with autoimmune thyroiditis (AIT), celiac disease, and pernicious anemia being the most common. Other less recognized associations, such as the co-occurrence of type 1 diabetes mellitus and multiple sclerosis, are also more frequent than might be thought, with studies reporting a threefold to fivefold higher prevalence of T1D in patients with MS. The exact mechanism behind this co-occurrence is not fully understood, but environmental factors (viral infections and vitamin D deficiency) and variations in non-human leucocyte antigen (HLA) class II alleles may be implicated. Understanding the similarities in the etiology and pathophysiology of these diseases may help clarify causality and create new strategies for the management of these conditions.

## Introduction

Type 1 diabetes mellitus (T1D) and multiple sclerosis (MS) are chronic diseases that result from the dysregulation of the immune system [1]. In T1D, there are specific autoantibodies directed against pancreatic β-cells implicated in the development of the disease [2,3]. On the other hand, in MS, the cause of chronic neurodegeneration remains unclear and there are no identified autoantibodies; there appears to be an inflammatory response mediated by autoreactive lymphocytes against specific molecules in the central nervous system [2,3].

Although MS and T1D are organ-specific autoimmune diseases, they share more etiologic and pathologic features than was expected between the two autoimmune diseases [2,3]. The prevalence of T1D in patients with MS seems to be greater in comparison to the general population [1,4,5]. However, the mechanism behind the co-occurrence of these diseases remains unclear. While genetic predisposition appears to be involved, the low concordance among identical twins for MS and T1D and trends of increasing incidence for both diseases over time suggest that environmental factors play an important role in the development of these diseases [4].

With this article, the authors intend to present the case of a patient with T1D who was later diagnosed with MS, alerting not only to the co-occurrence of these two autoimmune diseases but also to the diagnostic challenges that can arise while reviewing the etiologic similarities between them.

## Case presentation

We present the case of a 40-year-old Caucasian male, diagnosed with T1D at age 18. Since the diagnosis, he maintained surveillance with a diabetes care provider and was medicated with glargine and lispro insulin. He had poor metabolic control for his age, with a mean hemoglobin A1c (HbA1c) of 9.0%, and had a ketoacidosis episode at age 28. This might be explained by the fact that he only monitored his blood glucose levels two to three times a day and administered lower doses of fast-acting insulin than indicated because he reported fear of having hypoglycemia, which is known to be a major barrier to achieving glycemic control. His body mass index was normal (24.10 kg/m2) and he did not have any other known comorbidities; he denied smoking, alcohol, or recreational drug consumption. He had no family history of diabetes mellitus, neurodegenerative or autoimmune diseases.

At the time of diabetes diagnosis, the biochemical blood tests showed positivity for islet antigen 2 autoantibodies (98.1 UI/mL, reference <15 UI/mL) and negativity for glutamic acid decarboxylase (GAD) and insulin autoantibodies (zinc transporter 8 autoantibodies are not available in our hospital). During the follow-up, he maintained normal renal function with no evidence of microalbuminuria (<20 mg/24 hours); blood pressure and lipid levels were within the recommended targets without medication. He performed annual retinographies and had no diabetic retinopathy. He had normal thyroid function with positive anti-peroxidase autoantibodies (3220 UI/mL, reference <60 UI/mL) and negative antithyroglobulin autoantibodies, suggestive of autoimmune thyroiditis (AIT).

At age 32, 14 years after the diagnosis of T1D, he started complaining of paresthesia in both feet, worse during the night, and erectile dysfunction. Months later, he began to have episodes of muscle weakness and decreased strength in the right lower limb, with two to three weeks of evolution and complete resolution of the symptoms between crises. These symptoms were initially ignored by the patient, but when he presented with diplopia, he sought medical assistance from his diabetes care provider. He was referred to the emergency department where a brain computed tomography (CT) scan was performed, which excluded traumatic, vascular (ischemic or hemorrhagic), or space-occupying lesions, but showed hypodense subcortical white matter lesions in both cerebral hemispheres. He was discharged and the study was completed on an outpatient basis.

**Figure 1:**
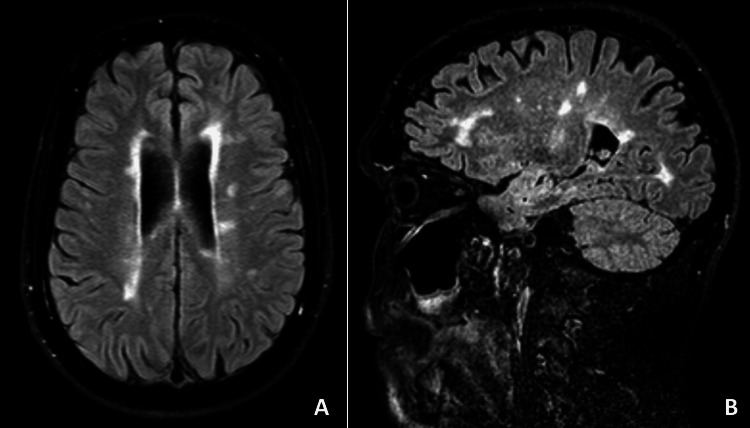
Brain magnetic resonance imaging These images show multiple infracentimetric lesions with hyperintense signals in fluid-attenuated inversion recovery (FLAIR) images, with evidence of space (multiple lesions in different locations) and time (lesions with different contrasts uptake) dissemination, suggestive of primary demyelinating disease: (A) axial (transverse) plane; (B) sagittal plane.

**Figure 2:**
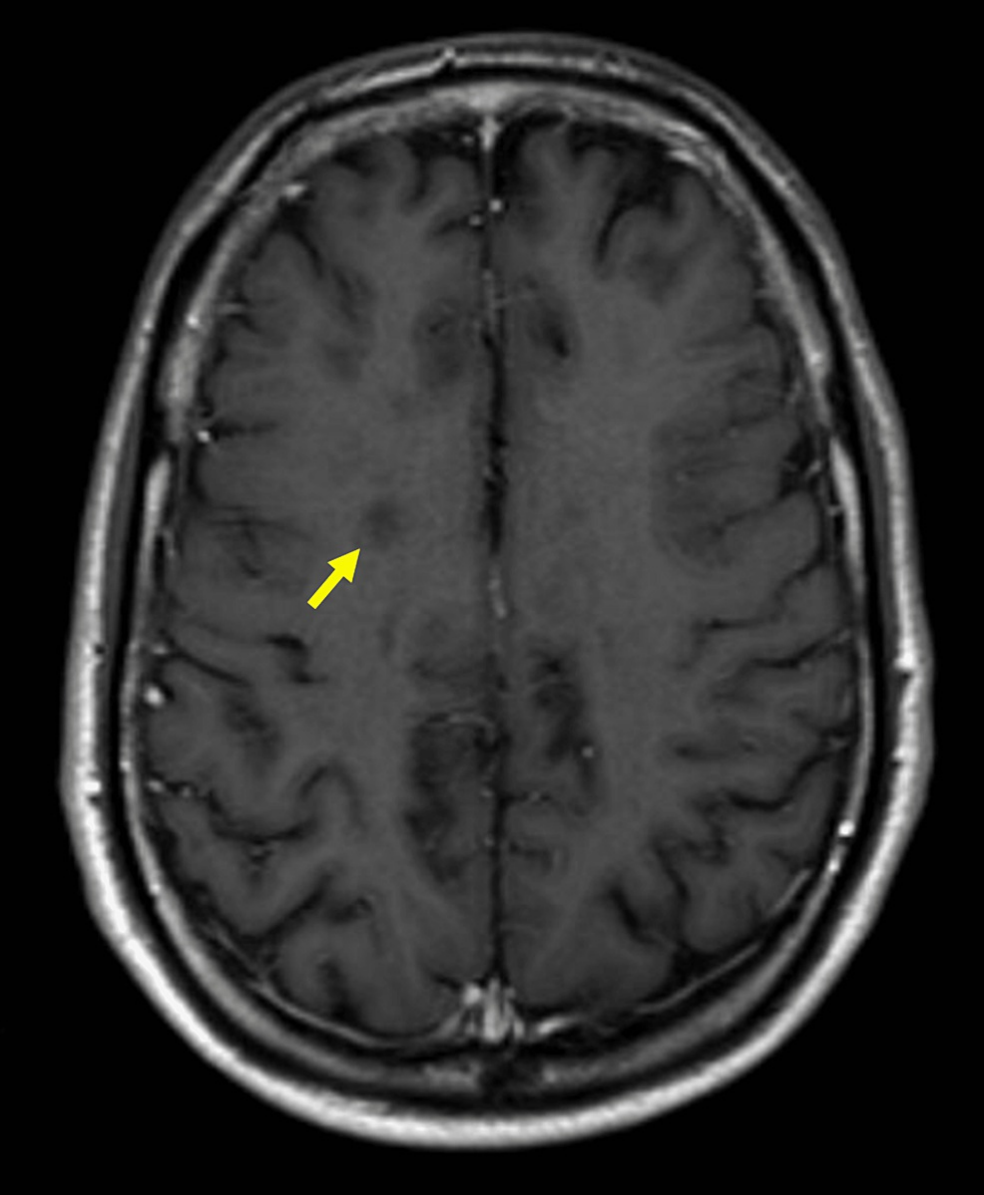
Brain magnetic resonance imaging This image shows hypointense lesions in the right centrum semioval, in T1-weighted image following administration of gadolinium, suggesting lesions with a necrotic center ("black holes" - arrow).

Electromyography was performed to characterize the neuromuscular function and showed signs of mild, chronic, sensory, and axonal polyneuropathy, with a disto-proximal gradient, suggestive of diabetic neuropathy, and non-specific signs of motor unit weak activation in the right lower limb muscles, which may be an indirect sign of central nervous system disorder. A brain magnetic resonance imaging (MRI), to better characterize CT scan findings, was requested which revealed multiple infracentimetric lesions, suggestive of primary demyelinating disease, with imaging evidence of space and time dissemination, and the diagnosis of MS was established (Figure 1A, 1B, 2). Cerebrospinal fluid analysis showed oligoclonal bands that sustained this diagnosis.

Multiple relevant investigations were also carried out to exclude other causes that could explain this clinical picture. Vitamin B12, folic acid or zinc deficiency, and Lyme disease were excluded (Tables 1-2). The blood count, kidney and liver function, plasma protein electrophoresis, immunoglobulins, sodium, potassium, chlorine, calcium, and magnesium levels were normal (Table 1); the autoimmune panel showed a non-specific, low antinuclear antibody (ANA) titre, celiac disease antibodies and human immunodeficiency virus (HIV), syphilis, and cytomegalovirus serologies were negative (Table 2).

**Table 1:** Biochemistry workup performed during the study of new onset neurologic symptoms AST: aspartate aminotransferase, ALT: alanine aminotransferase, TSH: thyroid-stimulating hormone.

Laboratory test	Results	Normal ranges
Hemoglobin	14.1	14.0–18.0 g/dL
White blood cells	5.50 × 10^9^	4.50–11.50 × 10^9^/L
Platelets	253 × 10^9^	150–450 × 10^9^/L
Blood urea	41	14–42 mg/dL
Serum creatinine	0.9	0.6–1.3 mg/dL
Serum sodium	142	136–145 mEq/L
Serum potassium	4.5	3.5–4.5 mEq/L
Serum chlorine	104.3	98.0–107.0 mEq/L
Serum calcium	4.9	4.2–5.1 mEq/L
Serum magnesium	0.84	0.53–1.11 mMol/L
Serum total proteins	7.8	6.6–8.7 g/dL
Serum albumin	4.6	3.5–5.0 g/dL
Alkaline phosphatase	68	25–100 Ul/L
AST	24	4–43 Ul/L
ALT	25	4–43 Ul/L
Immunoglobulin A	270.0	40.0–350.0 mg/dL
Immunoglobulin G	1076.0	650.0–1600.0 mg/dL
Immunoglobulin M	180.0	50.0–300.0 mg/dL
B12 vitamin	449.0	179.0–1130.0 pg/mL
Folic acid	11.8	1.0–20.0 ng/mL
Serum zinc	92	80–120 mcg/dL
Free T4	1.0	0.9–1.8 ng/dL
TSH	4.326	0.350–5.500 mUI/L

**Table 2:** Serology and autoimmune workup performed during the study of new onset neurologic symptoms

Laboratory test	Results
Antinuclear antibody	1/160 - nuclear dense fine speckled pattern
Anti-double stranded DNA antibody	Negative
Complement (C3 and C4)	Without consumption
ENA screen (SSA, SSB, Sm, RNP, Scl-70, Jo-1, centromere)	Negative
Borrelia burgdorferi antibodies	Negative
Anti-transglutaminase antibodies	Negative
Cytomegalovirus	Positive IgG antibodies with negative IgM antibodies, suggestive of previous contact
Treponema pallidum antibodies	Negative
HIV 1 and 2	Negative

He was referred for a neurology outpatient appointment and therapy with interferon β 1b was initiated. The disease progressed with a relapsing-remitting phenotype, with partial recovery between crises within days. He had two flares of the disease over the last eight years, requiring intravenous methylprednisolone pulses of 1000 mg daily for three to five days. He maintained biannual surveillance with his neurologist and performed an annual brain MRI. His last MRI showed increased lesion load with contrast-enhancing lesions, and he was proposed for therapy with ocrelizumab.

Because the patient maintained poor metabolic control, especially during the treatment of MS flares, he was proposed for continuous subcutaneous insulin infusion, which was placed in 2019. Since then, there has been a significant improvement in metabolic control, with a mean HbA1c of 7%. He maintains surveillance with a diabetes care provider every three months without evidence of other micro or macrovascular complications beyond diabetic neuropathy.

## Discussion

The prevalence of autoimmune diseases in the general population is approximately 4-5% [6]. T1D and MS represent a significant part of the autoimmune diseases that affect young adults and are an important public health problem worldwide [4].

People with T1D are at increased risk for other autoimmune diseases, with AIT, celiac disease, and pernicious anemia among the most common [7]. Given the high prevalence of these diseases in association with T1D, their screening is recommended in patients with T1D [7]. In this case, the patient had positive anti-peroxidase autoantibodies suggestive of AIT with normal thyroid function. However, other less recognized associations, such as the co-occurrence of T1D and MS, are also more frequent than might be thought.

Although diabetic neuropathy is the main cause of neurological symptoms in diabetic patients and may have a heterogeneous presentation [8], other neurologic diseases might be present. Physicians should be alert and early recognize neurologic symptoms that are not consistent with diabetic neuropathy so that an alternative diagnosis might be considered. In this case, we had a young patient with T1D with 14 years of evolution and poor metabolic control, which are strong risk factors for the development of diabetic neuropathy, and the electromyography was suggestive of this complication. However, new onset symptoms of decreased strength and diplopia and their relapsing-remitting course could not be explained by diabetic neuropathy, which alerted the authors to the hypothesis of other neurologic diseases, such as MS.

Multiple studies have demonstrated the association and shared etiopathogenesis between T1D and MS [4-6,9-11]. A Sardinia cohort study found a fivefold and twofold higher prevalence of T1D in patients with MS and their first-degree relatives, respectively, compared with the general population [6]. In a Danish cohort study, patients with T1D were at a threefold increased risk for the development of MS, and the risk for T1D in first-degree relatives of patients with MS was increased by approximately 40% [5]. A higher risk was found in an American study carried out in a population of women with T1D, who presented a 20-fold increased risk of developing MS [11].

The etiopathogenesis of this association is not yet fully understood. T1D and MS are T-cell-mediated disorders [1]. T-cells from patients with T1D show cross-reactivity against pancreatic islet and central nervous system antigens in vitro [12]. Similarly, peripheral blood from patients with MS contains T-cells that target islet and central nervous system antigens [12].

Genetic susceptibility for T1D and MS has been demonstrated in epidemiological studies [3]. Much of the genetic risk seems to be traced to the human leucocyte antigen (HLA) class II region [3]. However, sharing of haplotypes is unlikely, considering that the HLA haplotype DRB1*1501‐DQA1*0102‐B1*0602 confers susceptibility to MS but protects against T1D [1,3,9]. Therefore, the situation appears to be more complex than previously thought, and the risk for T1D and MS is probably not determined by a single HLA allele, but by the interaction between alleles of different loci [1,3]. Genome-wide association studies have discovered some single nucleotide polymorphisms (IL2RA, IL7R, CLEC16A, and CD226) involved in immunologic pathways that might have a modest effect on the co-occurrence of T1D and MS [3,13]. These findings, associated with the fact that concordance rates in monozygotic twins are considerably less than 100% in both T1D and MS [14,15], show that these are complex diseases, in which genetic predisposition, determined by multiple genes, interacts with environmental triggers to influence susceptibility to the disease.

MS and T1D share some environmental factors that are thought to be involved in their pathogenesis. A latitude gradient is present, with both diseases becoming increasingly common as the distance from the equator increases [3]. This gradient suggests that vitamin D may be an underlying factor in the development of these diseases [1,3].

The month of birth also seems to influence susceptibility to both diseases [3]. The risk of both diseases is higher in people born in the spring [3]. Vitamin D is the leading candidate for this seasonal effect, but infectious agents may also play a role [3].

Viral infections might trigger autoimmunity through the activation of different components of the immune system [16]. Enteroviruses, such as coxsackie virus in T1D and Epstein-Barr virus in MS, are considered possible triggers through mechanisms of molecular mimicry and cross-reactivity [3,16]. Besides this hypothesis, no causal relationship has been yet demonstrated between a virus infection and the development of any of these diseases [16]. Finally, vitamin D plays an important role in modulating inflammatory and immune functions. Some observational studies in MS and T1D have provided suggestive evidence that vitamin D supplementation results in a significantly decreased risk of these diseases [17,18]. The critical period during which the risk is determined is not clear, but some evidence suggests that it might be more dependent upon vitamin D deficiency in early life [3,17,18].

## Conclusions

Patients with one autoimmune disease are at increased risk for developing other autoimmune diseases, as is demonstrated in this case. The association between T1D and AIT is well recognized, but this case highlights other less recognized associations, such as the co-occurrence of T1D and MS, which is also more common than might be expected. The authors also intend to draw attention to the fact that a high level of suspicion is needed when neurologic symptoms appear in patients with T1D, especially when they are not typical of diabetic neuropathy, so alternative diagnoses might be investigated.

The exact mechanism behind the co-occurrence of T1D and MS is not fully understood. Further studies to better understand the similarities in etiology and pathophysiology between these diseases and how they might influence each other's clinical courses are important. They might provide new strategies for the management and prevention of these conditions that significantly affect quality of life.
